# Technology-assisted recovery of walking after spinal cord injury

**DOI:** 10.1007/s00415-019-09227-x

**Published:** 2019-02-13

**Authors:** Willis MD, Robertson NP

**Affiliations:** Institute of Psychological Medicine and Clinical Neuroscience, Cardiff University, University Hospital of Wales, Heath Park, Cardiff, CF14 4XN UK

Spinal cord injury (SCI) occurs most commonly as a result of trauma, although the proportion of cases related to non-traumatic aetiologies is rising over time. It has been estimated that the worldwide incidence of SCI lies between 250,000 and 500,000 per year. Injury to the spinal cord is devastating for patients; in addition to the loss of motor and sensory function, patients frequently require on-going management of bladder and bowel symptoms, pain, autonomic dysfunction and the psychological consequences of chronic disability. In higher income countries, access to rehabilitation and specialist medical care improves outcomes, but the ability to walk, or even stand again remains unachievable for many.

Currently activity-based therapies are the only medical intervention known to aid recovery, although patients without any voluntary muscle control receive minimal benefit. As a result, and to aid motor recovery after SCI, neurotechnology has been an important focus of research. The aim of such technologies is to enable active movement to enhance reorganisation of neuronal pathways and subsequent functional improvement. Of currently available technologies which have been examined in clinical trials, epidural electrical stimulation (EES) of the spinal cord is considered to have the most potential in achieving these goals. By stimulating proprioceptive circuits in the posterior roots of the spinal cord, EES activates motor neurone pools and augments residual, spared descending pathways under supraspinal control.

The three papers reviewed in this month’s journal club consider the role of EES in restoring over-ground walking in patients with SCI. Although these studies largely provide proof-of-concept, they are an important and encouraging forward step in the more widespread use of neurotechnology in patients with SCI. However, it is clear that further clinical trials will be necessary to validate results, particularly to identify which patients are most likely to benefit and at what stage of injury the intervention will be most efficacious. In the near future the cost of this technology coupled with intensive locomotor training (LT) is likely to be prohibitive for the majority and particularly for patients in low-income countries, but offer the vision of an optimistic future for affected patients.

## Recovery of over-ground walking after chronic motor complete spinal cord injury

This study investigated the use of stimulation alongside LT for recovery of standing and walking in patients with SCI. Four patients with traumatic, motor complete SCI (absence of voluntary movement below the level of injury with or without preserved sensation) were selected for the study.

The initial phase of the study involved intense LT on a treadmill with bodyweight support and manual facilitations of stepping. This was performed for 2 h, 5 days per week for 8–9 weeks. Following this, a 16-electrode epidural array was implanted over spinal segments L1 to S1-2 and a spinal cord stimulator inserted into the abdominal wall. Electromyography (EMG) was utilised to identify the desired combination of electrical stimulation, which would result in enhanced standing and stepping movements. Three types of training sessions were then performed with the stimulator turned on; (1) stepping on a treadmill, (2) over-ground standing and (3) over-ground walking. Each session lasted 1 h with 1–2 training sessions per day. Over-ground walking occurred only if the preceding skills were attained.

Following conventional rehabilitation, patients were unable to stand, walk or voluntarily move their legs. Study treatment was then commenced 2.5–3.3 years after the initial injury. The initial period of intense LT did not result in any changes to sensory, motor or locomotive ability. Following therapy with epidural stimulator assistance, two patients with American Spinal Injury Association scale (AIS) grade B (partially retained sensation) achieved the ability to walk over-ground with assistive devices. One of these patients (C5 level of injury) was able to walk over-ground while using horizontal poles or with bilateral assistance after 85 weeks of therapy with a maximum continuous distance of 90.5 m achieved. Independent standing with a walker and sitting for 5 min was also accomplished. Interestingly, independent walking was only possible when the patient intended to walk. The other patient (T1 level of injury) was able to walk independently with a walker after 15 weeks. Independent standing whilst holding onto elastic bands for approximately 50 min was also achieved. The other two patients, both with AIS grade A injuries (no movement or sensation below level of injury) were not able to perform over-ground walking but did achieve some components of independent stepping on the treadmill with body-weight support. They were also able to sit and stand independently. The main adverse outcome was a spontaneous hip fracture in one patient (AIS grade A) when stepping on the treadmill.

*Comment* This paper demonstrates the utility of epidural stimulation coupled with intensive LT therapy for patients with SCI. Particular benefit was observed in those patients with preserved sensation. Walking also only occurred when the intention to walk was present, which the authors speculate could be due to the activation of interneuronal networks in the lumbosacral cord. The results of this study are encouraging but will require replication in a larger group of patients to confirm efficacy and safety.

Angeli CA et al. NEJM. 2018;379(13):1244–1250.

## Neuromodulation of lumbosacral spinal networks enables independent stepping after complete paraplegia

Similar to the paper by Angeli et al. this paper focuses on the use of EES to facilitate over-ground walking in a single patient with chronic SCI.

A 26-year-old male patient who experienced a traumatic SCI 3 years prior, with complete loss of sensorimotor function below the 6th thoracic spinal segment was included in the study. Following injury, the patient underwent an initial 8 weeks of inpatient rehabilitation followed by 5 weeks of outpatient rehabilitation then 132 weeks without intervention. Despite clinical evidence of complete loss of function, imaging demonstrated intact tissue within the SCI site suggesting an incomplete injury.

Twenty-two weeks of LT was performed prior to implantation of a 16-electrode EES array on the dorsal surface of the lumbosacral cord, with a pulse generator implanted in the abdomen. Over 43 weeks, following EES insertion, 113 sessions of multi-modal rehabilitation (MMR) were performed. MMR sessions involved task-specific training in the presence of EES with activities performed while supine, side-lying, seated, standing and stepping with trainer assistance and body-weight support (BWS) as required. During this time, an additional 72 home-based exercise sessions with EES were also undertaken.

EES was optimised over the course of MMR with two interleaved EES programs found to enable bilateral control of leg function. Following initial outcomes of standing and intentional control of step-like leg movement using EES, the authors determined whether the patient could achieve independent stepping during 43 weeks of MMR. By week 43 of MMR, the patient achieved independent stepping on the treadmill at a speed of 0.22 m/s using arms on the support bars for balance without trainer assistance or BWS. At this stage, over-ground stepping was also achieved whilst using a front-wheeled walker and intermittent trainer assistance to allow weight shifting and to maintain balance. Throughout the study period, EES enabled improvement in step speed, with the maximum number of steps and distance travelled in a session being 331 and 102 m, respectively. At several time points throughout the study, clinical evaluation did not demonstrate any functional change with EES turned off.

*Comment* The use of task-specific EES in this study facilitated independent standing, stepping on a treadmill, and stepping over-ground with front-wheeled walker and intermittent trainer assistance. The authors speculate that MMR may have enhanced and reorganised existing spinal neuronal networks and preserved supraspinal-spinal connections associated with locomotor activities. As with the first paper discussed, although offering some optimism, the results of this study will need to be replicated in a larger clinical trial to confirm efficacy.

Gill ML et al. Nat Med. 2018;24(11):1677–1682.

## Targeted neurotechnology restores walking in humans with spinal cord injury

This paper reports the use of spatiotemporal EES during over-ground LT training in humans with chronic SCI. The authors hypothesised that this technology could enable voluntary locomotion, leading to functional improvements with and without EES stimulation.

Three male patients were included in the study, each with a chronic unspecified cervical SCI and AIS scale C-D (motor incomplete). Inclusion criteria for the study specified the ability to stand with a walker or 2 crutches. Two of the patients were classified as being able to walk 10 metres with varying levels of assistance before intervention. A 16-electrode EES array was implanted to target the lumbo-sacral nerve roots that projected to spinal cord regions associated with motor neuron activation for the hip, knee and ankle joints necessary for locomotion. An implantable pulse generator with wireless communication then enabled real-time control over EES parameters during over-ground walking. This was aided by a recording platform providing real-time processing of whole-body kinematics, ground reaction forces and EMG of leg muscles. A multidirectional gravity-assist device provided BWS.

Once optimised, patients regulated the timing of their movements to pre-programmed EES sequences. This enabled EMG activity during stepping on a treadmill within 5 days, followed by all patients walking voluntarily over-ground with EES stimulation. Following this, patients were able to adjust their range of leg movements, stride speed and length and able to stop movements despite ongoing stimulation. Overall, patients were able to walk on a treadmill for 1 h with a maximum achieved distance of 1 km. Following an intensive, 5 month, EES-enabled rehabilitation program, all patients improved their walking capacities. Strikingly, improvements to walking were also observed when EES was not in use. Interestingly, when continuous EES was utilised, as in the first two studies reviewed here, the authors found this to be ineffective. In addition to over-ground walking, the authors also engineered a tablet-based system whereby EES sequences could be selected with a voice-controlled watch to facilitate standing, walking or cycling. To enable this, EES sequence algorithms were triggered and adjusted based on real-time acquisition of signals from wearable inertial measurement units.

*Comment* This study is remarkable for the restoration of walking in patients with SCI. As opposed to empirical, continuous stimulation used in the aforementioned studies, the use of spatiotemporal EES sequences coinciding with the intended movement allowed more natural control of leg movements. The authors hypothesise that through plasticity this increases the strength and number of spared descending pathways and is responsible for the recovery observed without EES. It is important to note that all patients had motor incomplete SCI and, therefore, the results may not extrapolate to those with more severe injury. In addition, this is a proof-of-concept study and, therefore, further clinical trials will be necessary to assess its true impact.

Wagner FB et al. Nature. 2018;563(7729):65–71.

